# Outcomes of Structured Medication Reviews for Selected Patients in the English National Health Service †

**DOI:** 10.3390/pharmacy13050142

**Published:** 2025-10-01

**Authors:** Michael Wilcock, Marco Motta, Chris Burgin

**Affiliations:** Medicines Optimisation Team, NHS Cornwall and Isles of Scilly Integrated Care Board, Beacon Technology Park, Part 2S, Chy Trevail, Dunmere Rd., Bodmin PL31 2FR, UK; mike.wilcock@nhs.net (M.W.); marco.motta@nhs.net (M.M.)

**Keywords:** medication review, primary care, polypharmacy, addictive medication, general practice

## Abstract

Structured medication reviews are now a common part of primary care practice, but little information is available on the outcomes of these reviews. We incentivised practices to submit a report via MS Forms supplying information from and the outcomes of the reviews of two cohorts of patients (those prescribed potentially addictive medication and those on problematic polypharmacy), as defined in the Primary Care Networks Contract Directed Enhanced Service. Submissions were analysed in Microsoft Excel. By the end of March 2025, 2858 reports were received from 48 of 55 eligible practices, reviewing a total of 34,531 prescribed items, with a mean of 12.1 items reviewed per structured medication review. Results indicated a preference amongst patients for the remote delivery of reviews, though changes to prescribed medication were more common following face-to-face contact. A total of 2706 changes to prescribed medication were made at a mean rate of 0.9 per structured medication review, with pain management being the most common British National Formulary category altered, though this may be because of the cohorts chosen. The most common change across all reviews was the discontinuation of a prescribed item. In reviews for the potentially addictive medication cohort, a reduction was proposed and accepted in 43.5% of cases. Additional interventions, which took place in 83.9% of reviews, were also captured.

## 1. Introduction

The prescribing of medicines is the most widely used medical intervention. However, harm caused by medicines is a recognised problem, and the World Health Organization’s Third Global Patient Safety Challenge: Medication Without Harm aimed to reduce the global level of severe, avoidable harm related to medications by 50% between 2017 and 2022 [1]. The negative consequences of overprescribing in England were described in The Department of Health and Social Care’s National Overprescribing Review [2]. This report noted that 8.4 million patients regularly take five or more medicines; one in five hospital admissions in patients aged over 65 result from adverse drug effects; the risk of harm increases with the number of medicines taken; and 10% of prescriptions in primary care are inappropriate, with alternative treatments often better for a patient’s needs and preferences [2].

Medication review (MR) is recommended as a means of reducing preventable adverse drug events and hospital admissions [3]. The origins, variety, types, and historical background of MRs have been well described from an international perspective [4]. This review notes that an MR should be carried out by a skilled healthcare professional, e.g., pharmacist, general practitioner (GP), or nurse, with interprofessional collaboration being a key element of the MR process. The authors comment that there are no globally recognised conclusive standards for the identification of patients to be prioritised for MRs. However, MRs typically target factors correlated with drug-related problems including multimorbidity, the complexity of the medication schedule, the patients’ age and frailty, and the presence of high-risk medicines. A reduction in inappropriate prescriptions, a reduction in drug-related problems, and increased adherence are deemed to be consistent positive effects of MRs [3], though others point to MRs having minimal effect on clinical outcomes [5,6,7]. Furthermore, in relation to polypharmacy, a review of systematic reviews found that interventions to reduce problematic polypharmacy are effective, but there is no evidence on clinically relevant patient outcomes [8].

MRs in the English primary care setting have previously been promoted as part of the Quality and Outcomes Framework. Structured medication reviews (SMRs), mainly led by clinical pharmacists, have been conducted under the Primary Care Networks (PCNs) Contract Directed Enhanced Service (DES) since 2020 as part of a national priority to improve medicine optimisation and reduce polypharmacy [9]. PCNs provide a formal structure for a geographically based network of general practices covering populations of 30–50,000 patients. The DES contract, which sets out the core requirements of and entitlements for a PCN, appropriately highlights trained clinical pharmacists as the professionals who should take responsibility for the delivery of SMRs, including for care home residents. The purpose and objectives of SMRs have been described by the National Institute for Health and Care Excellence [10]. An SMR is a comprehensive review of a patient’s medication, focusing on tailoring care to their unique needs, preferences, and circumstances. They are intended to improve the quality of prescribing, delivering improvements to patient care and outcomes [11]. One of the key aspects to the delivery of SMRs is to address complex or problematic polypharmacy and reduce the risk of adverse effects. Problematic polypharmacy, or inappropriate polypharmacy, occurs when medicines are no longer appropriate; benefits do not outweigh harms; combinations cause or risk harm; and usage becomes unmanageable or distressing. SMRs may be undertaken face-to-face or remotely where deemed clinically appropriate. However, as with MRs in general, published evaluations of SMRs are limited in number and scope [12,13].

This study aims to gather information on the prescribing outcomes of SMRs across one Integrated Care System (ICS) in the southwest of England, with a focus on SMRs for patients on complex and problematic polypharmacy (PP), and those taking potentially addictive medications (PAMs). An ICS is a local partnership that brings health and care organisations, including the voluntary sector, social care providers, and other partners, together to develop shared plans and joined-up services.

## 2. Materials and Methods

### 2.1. Study Design and Setting

The project was prospectively designed to evaluate the outcomes from SMRs undertaken in general practices in one geography in the southwest of England. This project was part of the 2024/25 Cornwall and Isles of Scilly Integrated Care System (ICS) medicine optimisation incentive scheme. There was no requirement for formal ethics committee approval under the Health Research Authority criteria. Furthermore, no patient details (age, gender) were collected.

All 55 practices were asked to submit the outcomes of SMRs conducted between 1 April 2024 and 31 March 2025 for patients falling into the PAM or PP cohorts, as defined in the DES. The DES refers to clinical medication reviews for people with complex polypharmacy (specifically those on 10 or more medications), especially older people, people in care homes, those with multiple co-morbidities, and people with learning disabilities or autism. However, we chose our two areas because we wanted to provide an incentive where practices would be focusing on the same complexity of patient cohorts, reducing inequality and providing equity of time needed for the review. Where a patient fell into both cohorts, respondents were asked to select PAM to trigger the questions relating to dose reduction. Though the DES refers to patients requiring SMRs and follow-up consultations, we sought data on patients having a unique SMR in the time period described.

Payment was available to practices that submitted a total number of reports at least equal to 0.5% of their registered list size; that is, the number of patients registered with the practice. As an example, for a typical practice of 10,000 patients, the payment for submitting 50 reports would be approximately GBP 1500. This value of 0.5% of the list size was chosen such that small and large-sized practices would be able to identify sufficient patients to be reviewed. The actual payment, as described above, is similar to the payment that was available to practices for other elements of the medicine optimisation incentive scheme.

### 2.2. Data Collection

A self-administered online form (Microsoft Forms) was specifically developed for this study. Outcome reports were submitted by the person undertaking the SMR through this form for ease of reporting and subsequent analysis in Microsoft Excel 365. Conditional questions (which allowed the respondent to skip certain questions based on previous answers) were used to keep the form length to a minimum. Information on reviewed prescription items was limited to the British National Formulary (BNF) category to simplify reporting [14]. The BNF is a United Kingdom (UK) pharmaceutical reference book containing information and advice on prescribing, pharmacology, and specific facts and details about many medicines available on the UK National Health Service (NHS). Questions were limited to option selection as much as possible to reduce variation in the data, with a free-text section at the end for the respondent to supply any further information they considered useful. No patient identifiable data was to be submitted. See Supplement A for the questions and options used.

Microsoft Forms outputs the responses to an Excel spreadsheet from which basic analyses such as filtering and calculating totals, percentages, and mean averages was performed. Practices were not asked to submit new or updated reports where there were further changes to a patient’s medication beyond those agreed to during the SMR.

## 3. Results

**Figure 1:**
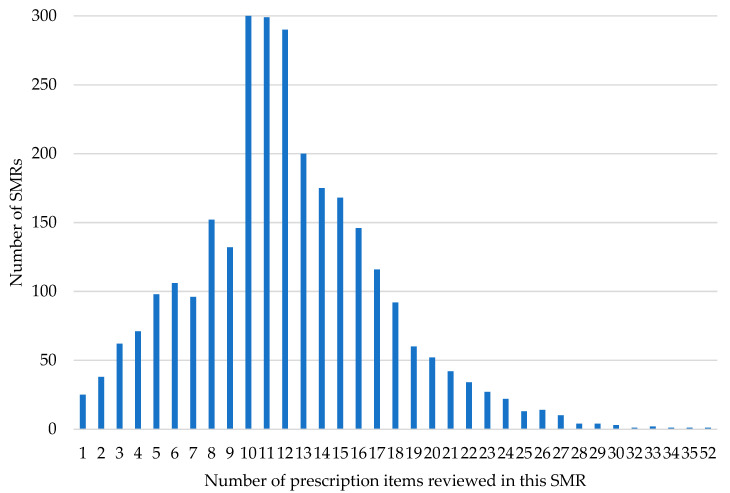
Number of prescription items reviewed in each SMR.

By the end of March 2025, 2858 reports were received from 48 of 55 eligible practices, reviewing a total of 34,531 prescribed items, with a mean of 12.1 items reviewed per SMR (IQR = 6). The mean number of prescribed items per patient was 13.7 for the polypharmacy cohort and 10.1 for the PAM cohort. As noted in 2.1, patients that fall into both cohorts were recorded as part of the PAM group. The distribution of items per SMR is illustrated in Figure 1. Of the patients receiving an SMR, 44.2% (1263) were selected due to taking potentially addictive medication (PAM), and 55.8% (*n* = 1595) were selected due to problematic polypharmacy (PP).

### 3.1. Mechanism of Delivery

Remote delivery of all SMRs accounted for 85.9% (2454/2858) of all reports; 84.8% (1071/1263) of PAM SMRs and 86.7% (1383/1595) of PP SMRs were delivered remotely. The delivery method of the SMR was in accordance with the patient’s choice for 84.8% (2080/2454) of remote consultations and 95.1% (384/404) of face-to-face consultations.

### 3.2. Changes to Prescribed Medication

A total of 2706 changes to medication were made, with a mean of 0.9 per SMR. One or more changes to prescribed medication were made in 60% (1714) of SMRs. Medication changes were reported to occur more frequently following a face-to-face SMR (73.5%) than via a remote SMR (57.7%, Table 1). Advice and counselling were given for an additional 1943 unchanged prescription items.

Table 2 summarises the number of changes made to prescribed medication by BNF category. The greatest number of changes were made to prescriptions for pain management (26%, 703), followed by gastrointestinal system medication (14.7%, 397), other cardiovascular medicines (14%, 378), and other central nervous system medications (12.8%, 346).

A total of 39.1% (1057) of changes resulted in a prescribed item being discontinued. Dose changes accounted for 36.1% (978) of all alterations to prescriptions (Table 3).

For a full breakdown of the types of changes made to each BNF category, see Table S1. For splits by SMR cohort, see Tables S2 and S3.

### 3.3. PAM SMRs and Dose Reduction

The potentially addictive medication(s) being prescribed, as reported in each SMR, are summarised in Table 4. A total of 25.7% of patients were prescribed two or more such medicines at the time of the review.

A reduction in PAM was suggested in 82% (1036/1263) of PAM SMRs and accepted by the patient in 53% (549/1036) of those SMRs where it was suggested.

Overall, 43.5% (549/1263) of all PAM SMRs had a reduction proposed and accepted.

- A total of 46.9% (90) of face-to-face PAM SMRs had a reduction proposed and accepted.
- A total of 42.9% (459) of remote PAM SRMs had a reduction proposed and accepted.

### 3.4. Other Interventions

Respondents were asked to record any other inventions made from a list provided, and for 2396 (83.9%) SMRs there was at least one intervention documented. Note that each patient may have more than one of these recorded in the same SMR. The nature and number of these interventions (a total of 4829 instances) are shown in Table 5.

### 3.5. Notable Comments from Respondents

A free-text section at the end of the reporting form was available for the clinician to add any additional details or record outcomes not captured in the preceding questions. Of the 2858 reports, free-text comments were entered in 588 (20.1%) reports. Common themes included notes on PAM reduction, deprescribing, issues relating to patient understanding and compliance, and setting up or acting on the results of monitoring. A selection of these responses as written is shown in Box 1.

Box 1 **Illustrative free-text comments received in the reports.**

NSAID stopped in view of newly found raised pro-BNP—awaiting input from GP, flagged on for GP review.

 

Patient overusing pregabalin! Risks etc discussed and stable dose agreed, + 2-weekly prescriptions.

 

Dapagliflozin initiated for heart failure with full medication counselling, furosemide dose accordingly reduced. Sick day rules discussed.

 

Codeine weaning plan agreed with patient, dose reduced by 50% currently.

 

Anticholinergic burden score reduced from 6 to 1. Amitriptyline stopped due to drowsiness side effects and Nefopam stopped as ineffective.

 

Medication screen updated with regards to quantities of medicines. Recent hospital stay so confirmation of medication as patient confused by some factors and had concerns about which medication to continue.

 

Using GTN daily and at night. Urgent electrocardiogram) and referral to cardiology. Isosorbide mononitrate and statin increased.

 

Spironolactone reduced due to raised K+ (5.7), follow-up arranged.

 

70y/o. Stopped Alendronic Acid, Calcium, iron tablets no longer needed. Counselled on dietary measures, falls preventions and lifestyle.

 

Poor compliance, only taking apixaban OM, changed to rivaroxaban OD, confusion with changes in diabetic meds & stopped taking all together. Medicine administration chart done to help improve understanding.

 

## 4. Discussion

These 2858 SMR reports totalled 2706 changes to prescribed medication, a mean of just under 1 change (0.9) to prescribed medication per SMR. Items stopped and doses decreased accounted for 62% (1688/2706)) of these changes. With a mean of 12.1 items per SMR, this reduction of 0.6 items (a 4.9% decrease) lies within the estimates of a percentage reduction in per-patient medicines ranging from 2.7% to 9.9% in the SMR study from Abuzour and colleagues [15]. The reduction we report aligns with tackling overprescribing and the benefit that this brings to the patient and the whole health system. Of the 2858 SMRs reported to us, 1144 (40%) recorded no change to medicines. A nationwide observational cohort study of patients aged ≥65 years who had received SMRs in the first two years of the programme reported that the majority of patients had no changes in their treatment following an SMR [16]. This larger study also noted that antihypertensives, cardiovascular medications, psychotropics, laxatives, and PPIs were the main classes of medicines stopped following an SMR [16]. Wood and colleagues reported on the percentage of patients with a medication review coded in an English general practice between April 2019–March 2022 and found that those prescribed potentially addictive medication showed the highest percentage of SMRs completed within the previous 12 months [17]. In particular, 575 (45.5%) of the 1263 PAM SMRs reported in our study were directed at opioid prescribing. Opioid drugs were the most prescribed dependency-forming medicine (from opioid pain medicine, gabapentinoids, benzodiazepines, and Z-drugs) across primary care in England in 2023/24 [18]. Others have described the importance of pharmacists (in both GP practices as well as those working in a hospital) leading medication reviews for effective opioid management [19]. It is unsurprising that in our study we did see that the greatest number of changes were made to prescriptions for pain management (26%, 703), as this cohort of patients (on PAM) was a target group for the SMRs.

Although the mean of 0.6 items per SMR stopped or the dose decreased is not high, the savings across the whole healthcare system for each patient is greater than the ‘cost’ of a single drug stopped, considering the optimisation of treatment and the review of the clinical profile of the patient (including referrals, new blood tests, and lifestyle/holistic advice given). Though a MR is a complex task, it has been shown that person-centred medicine reviews by general practice pharmacists for patients at a high risk of medicine-related harm provide substantial cost savings [20].

Lifestyle advice was provided in just under half (45%) of SMRs, linking with a simple person-centred care focus based on an individual’s needs, preferences, and values. Whilst for 9% of SMRs, the intervention was to refer the patient to services outside of the practice, pointing to an opportunity for integrated services and a wider, streamlined care.

In our study, the remote delivery of SMRs accounted for 85.9% of all SMRs. Though the majority of SMRs were undertaken remotely, it is acknowledged that the patient safety aspects of virtual consultations are underdeveloped [21]. We note that in a randomised trial delivering a patient-centred polypharmacy medication review in primary care, 74.6% were by phone consultations, and 79.9% of respondents reported they were happy with where the review took place [22].

Free-text comments were entered for approximately one in every five reports. Often, particularly where it appears the clinician has copied anonymised information from the clinical record of the SMR into the reporting form, these comments covered more than one theme incorporating discussions with the patient regarding their understanding of their medication, the need for monitoring, responding to patient concerns, dose adjustments, and more. Such comments reflect the complexity of SMRs and the variety of factors that need to be taken into consideration to provide a thorough review that benefits the patient. Details in several of these comments, combined with the number of interventions recording lifestyle advice, demonstrate that there is a focus on holistic care rather than just prescribed medication.

At the time of this work, there were approximately 11,500 patients prescribed 10 or more unique medicines across the ICS, and hence this scheme continues into 2025/26, though with a focus on different cohorts of patients.

This model of incentive scheme, ran in one county in the southwest of England, could be replicated nationwide, providing stronger evidence of these reviews by highlighting the multiple interventions and complexities that PCN pharmacy teams need to deal with in tackling inappropriate prescriptions and enhancing better care for the patients.

### Study Limitations

Limitations of this work include no information collected on patient outcomes other than medication changes and additional actions; hence, we are reliant on the accuracy and completeness of those filling in the survey form. We do not know to what extent non-medication interventions (Table 5) were followed-up and actioned. The self-reported survey form may not fully capture the complexity of conducting SMRs, as described elsewhere [15], and their outcomes with potential recall and social desirability biases. Our study was over a specific time period, and the scheme may have incentivised the SMRs to be undertaken. The actual number of SMRs provided is limited by the clinical pharmacist capacity of PCNs on which we do not report. We did not record which healthcare professional (GP or pharmacist) conducted the SMR, though our view is that the main pharmacists are undertaking these SMRs. Existing guidelines for SMRs in England emphasise that these reviews should be conducted using the principles of shared decision-making to underpin conversations with patients. The reports we received back from practices were not designed to collect aspects of the SDM process. Others have observed that, in the early years of the introduction of SMRs, implementation did not match the vision for patients presented in the policy of an invited, holistic, and shared decision-making opportunity offered by well-trained pharmacists [23]. It has been suggested that improving patient awareness about the role of the growing clinical pharmacist workforce may lead to a better patient experience in the context of polypharmacy medication reviews [22]. Drug changes were recorded only by BNF category to simplify reporting and analysis. The PAM cohort includes PP patients, which limits the analysis of PP outcomes. Furthermore, this was a small survey in one geographical region of the UK (Cornwall), and therefore, our results may not be generalizable.

## 5. Conclusions

This study provides insights into the implementation of a reporting system for SMRs in one Integrated Care System (ICS) in the southwest of England. The outcomes demonstrate how SMRs are a potentially valuable means of improving the use of medicines with the aim of improving patient outcomes and supporting the whole healthcare system.

## Figures and Tables

**Table 1:** SMRs where medication changes occurred.

	Face-to-Face	Remote	Total
PAM	73.4% (141/192)	60.5% (648/1071)	62.5% (789/1263)
PP	73.6% (156/212)	55.6% (769/1383)	58% (925/1595)
Total	73.5% (297/404)	57.7% (1417/2454)	60% (1714/2858)

**Table 2:** Changes to prescribed medication by BNF category.

BNF Category	Total	% of Changes
Pain management	703	26%
Gastrointestinal system	397	14.7%
Other cardiovascular medicines	378	14%
Other central nervous system medicines	346	12.8%
Nutrition and blood	156	5.8%
Antihypertensives	151	5.6%
Respiratory	111	4.1%
Obstetrics, gynaecology, and urinary tract	85	3.1%
Skin	83	3.1%
Antidiabetic drugs	79	2.9%
Musculoskeletal and joint diseases	52	1.9%
Ear, nose, and throat	42	1.6%
Eyes	37	1.4%
Other endocrine	31	1.2%
Appliances and dressings	22	0.8%
Infections	16	0.6%
Thyroid and antithyroid	9	0.3%
Immunosuppression	8	0.3%
Total	2706	100.0%

**Table 3:** Prescription change by type of change made.

BNF Category	Total	% of Changes
Dose decreased	631	23.3%
Item stopped—inappropriate	384	14.2%
Item stopped—patient not using/taking	388	14.3%
Item stopped—ineffective	141	5.2%
Item stopped—side effects	144	5.3%
New item prescribed	522	19.3%
Dose increased	347	12.8%
Swapped to direct equivalent (e.g., pMDI to DPI)	149	5.5%
Total	2706	100%

**Table 4:** Potentially addictive medicine(s) reviewed in PAM SMRs.

Potentially Addictive Medicine(s)	Patient Count
Opioid (monotherapy)	575 (45.5%)
Gabapentinoid (monotherapy)	236 (18.7%)
Benzodiazepine (monotherapy)	81 (6.4%)
Z-drug (monotherapy)	46 (3.6%)
Combination of 2 of the above	270 * (21.4%)
Combination of 3 of the above	51 (4%)
Combination of all 4 of the above	4 (0.3%)
Total	1263 (100%)

* A total of 183 of these patients were taking a combination of gabapentinoids and opioids.

**Table 5:** Type and number of interventions undertaken during SMRs.

Intervention	Number of Patients
Lifestyle advice given	1275
Monitoring organised for existing medication	1112
Updated patient information on medical record (e.g., weight, BP)	870
Patient escalated to GP	475
None of these	462
Biochemical test monitoring organised for new or changed medication	378
Patient referred to other HCP (e.g., We Are With You, Healthy Cornwall, social prescriber, pulmonary rehabilitation)	197
Patient escalated to secondary care via the GP	60
Total	4829

## Data Availability

The original contributions presented in this study are included in the article/Supplementary Material. Further inquiries can be directed to the corresponding author.

## Supplementary Materials

The following supporting information can be downloaded at: https://www.mdpi.com/article/10.3390/pharmacy13050142/s1, Supplement A: Microsoft Forms questions; Supplement B: Table S1: Changes to medication reported from all SMRs; Table S2: Changes to prescribed medication reported from PP SMRs; and Table S3: Changes to prescribed medication reported from PAM SMRs.

## Abbreviations

The following abbreviations are used in this manuscript:

MR	Medication review
SMR	Structured medication review
PCN	Primary care network
DES	Directed enhanced service
PAM	Potentially addictive medication
PP	Problematic polypharmacy
pMDI	Pressurized metered dose inhaler
DPI	Dry powder inhaler
NSAID	Non-steroidal anti-inflammatory drug
GTN	Glyceryl trinitrate
BNP	B-type natriuretic peptide
