# Supplementary material for: Outcomes of Structured Medication Reviews for Selected Patients in the English National Health Service
Source: Pharmacy (Basel). 2025 Oct 1;13(5):142. doi: 10.3390/pharmacy13050142 (PMC12566740; doi:10.3390/pharmacy13050142)
Supplement: Supplementary file 1 [file pharmacy-13-00142-s001.zip › pharmacy-3845893-supplementary.pdf]

Supplement A. Microsoft Forms questions

The questions and options from the MS Form used to gather information about SMRs and their outcomes

1. Practice ODS code
Free text entry

2. How was the SMR conducted
Choose one of:
- Face to face
- Remote

3. Was this the patient's choice?
Choose one of:
- Yes
- No

4. Was this patient chosen due to:
Choose one of:
- Potentially addictive medication
- Problematic polypharmacy

5. Which potentially addictive medicines were identified?
Conditional question only shown if answer to Q4 is "potentially addictive medication", select all that apply
- Opioid
- Gabapentinoid
- Benzodiazepine
- Z-drugs

6. Was a reduction in the potentially addictive medicines(s) discussed?
Conditional question only shown if answer to Q4 is "potentially addictive medication", choose one of:
- Yes
- No

7. Did the patient agree to try a reduction?
Conditional question only shown if answer to Q6 is "yes", choose one of:
- Yes
- No

8. Total number of prescribed items reviewed in this SMR
Numerical entry

9. Were any changes to medicines made as a result of this SMR?
Choose one of:
- Yes (go to next)
- No (go to Q 13)

10. Which therapeutic area does this change relate to?
Select from drop down:
- Gastrointestinal system

- Antihypertensives
- Other cardiovascular medicines
- Respiratory
- Pain management
- Central nervous system
- Infections
- Antidiabetic drugs
- Thyroid & antithyroid
- Other endocrine
- Obs, gynae & urinary tract
- Immunosuppression
- Nutrition & blood
- Musculoskeletal & joint diseases
- Eye
- Ear, nose & throat
- Skin
- Appliances and dressings

11. What type of change was made?
Select one from list:

- Item stopped as inappropriate
- Item stopped as ineffective
- Item stopped due to side effects
- Item stopped as patient not using/taking
- Dose increased
- Dose decreased
- Swapped to direct equivalent (e.g. pMDI to DPI)
- New medicine prescribed

12. Record another change to medication from this SMR?
Choose one of:

- Yes (further instances of Q7, 8 and 9 for total of 5 changes only shown if needed)
- No (go to next)

13. Select any therapeutic areas covered in the SMR where one or more items were not changed
Include any areas where some but not all items were changed
Choose all that apply:

- Gastrointestinal system
- Antihypertensives
- Other cardiovascular medicines
- Respiratory
- Pain management
- Central nervous system
- Infections
- Antidiabetic drugs
- Thyroid & antithyroid
- Other endocrine
- Obs, gynae & urinary tract
- Immunosuppression
- Nutrition & blood
- Musculoskeletal & joint diseases

- Eye
- Ear, nose & throat
- Skin
- Appliances and dressings

14. How many unchanged medicines did the patient receive advice and counselling for?
(Examples include counselling in line with MHRA alerts, inhaler technique, steroid card issued)
Numerical entry

15. Which of these other interventions were made as a result of this SMR?
Presented once, select all that apply

- Patient information updated (e.g. weight, BP)
- Monitoring arranged for existing medication(s)
- Monitoring arranged for new or changed medication
- Patient escalated to GP
- Patient escalated to secondary care
- Patient referred to other HCP (e.g. We Are With You, Healthy Cornwall, social prescriber, pulmonary rehabilitation)
- Lifestyle advice given
- None of these
-

16. Use this space to add any additional details or record outcomes not captured in the questions above
Free text entry, permit long answer

Supplement B

Table S1. Changes to medication reported from all SMRs

BNF Category	Dose decreased	Dose increased	Item stopped - inappropriate	Item stopped - ineffective	Item stopped - patient not using/taking	Item stopped - side effects	New item prescribed	Swapped to direct equivalent (e.g. pMDI to DPI)	Total
Antidiabetic drugs	10	11	10		14	8	16	10	79
Antihypertensives	19	44	20	1	12	26	24	5	151
Appliances and dressings	1		4		15		2		22
Ear, nose & throat	2	2	6	5	17	2	7	1	42
Eye			4	2	16	1	10	4	37
Gastrointestinal system	109	37	59	20	58	6	90	18	397
Immunosuppression	3	1			3		1		8
Infections			8	1	2	1	4		16
Musculoskeletal and joint diseases	7		12		7	6	14	6	52
Nutrition & blood	14	2	69	4	25	6	28	8	156
Obstetrics, gynaecology & urinary tract	4	11	7	20	20	7	12	4	85
Other cardiovascular medicines	50	71	54	6	39	41	85	32	378
Other central nervous system	97	74	29	23	45	11	55	12	346
Other endocrine	4	2	7	1	5	2	7	3	31
Pain management	296	72	71	48	65	20	107	24	703
Respiratory	10	14	11	8	15	6	33	14	111
Skin	2	3	12	2	29	1	27	7	83
Thyroid & antithyroid	3	3	1		1			1	9
Total	631	347	384	141	388	144	522	149	2,706

Table S2. Changes to prescribed medication reported from PP SMRs

BNF Category	Dose decreased	Dose increased	Item stopped - inappropriate	Item stopped - ineffective	Item stopped - patient not using/taking	Item stopped - side effects	New item prescribed	Swapped to direct equivalent (e.g. pMDI to DPI)	Total
Antidiabetic drugs	10	6	4		13	6	12	7	58
Antihypertensives	15	34	19		8	21	18	3	118
Appliances and dressings			3		8		2		13
Ear, nose & throat	1	1	6	3	13	2	7	1	34
Eye			1	1	15	1	7	3	28
Gastrointestinal system	60	24	40	14	34	5	50	12	239
Immunosuppression	1	1			1		1		4
Infections			3		1		4		8
Musculoskeletal and joint diseases	5		7		4	2	7	2	27
Nutrition & blood	9	2	51	3	16	4	17	6	108
Obstetrics, gynaecology & urinary tract	2	4	4	14	12	5	5	2	48
Other cardiovascular medicines	44	60	39	5	31	29	58	25	291
Other central nervous system	38	29	16	10	25	2	23	6	149
Other endocrine	3	2	6	1	3	2	5	3	25
Pain management	58	17	21	15	30	4	42	8	195
Respiratory	6	7	8	3	13	4	12	9	62
Skin	1	2	8	2	18		18	6	55
Thyroid & antithyroid		3	1		1				5
Total	253	192	237	71	246	87	288	93	1,467

Table S3. Changes to prescribed medication reported from PAM SMRs

BNF Category	Dose decreased	Dose increased	Item stopped - inappropriate	Item stopped - ineffective	Item stopped - patient not using/taking	Item stopped - side effects	New item prescribed	Swapped to direct equivalent (e.g. pMDI to DPI)	Total
Pain management	238	55	50	33	35	16	65	16	508
Other central nervous system	59	45	13	13	20	9	32	6	197
Gastrointestinal system	49	13	19	6	24	1	40	6	158
Other cardiovascular medicines	6	11	15	1	8	12	27	7	87
Respiratory	4	7	3	5	2	2	21	5	49
Nutrition & blood	5		18	1	9	2	11	2	48
Obstetrics, gynaecology & urinary tract	2	7	3	6	8	2	7	2	37
Antihypertensives	4	10	1	1	4	5	6	2	33
Skin	1	1	4		11	1	9	1	28
Musculoskeletal and joint diseases	2		5		3	4	7	4	25
Antidiabetic drugs		5	6		1	2	4	3	21
Appliances and dressings	1		1		7				9
Eye			3	1	1		3	1	9
Ear, nose & throat	1	1		2	4				8
Infections			5	1	1	1			8
Other endocrine	1		1		2		2		6
Thyroid & antithyroid	3							1	4
Immunosuppression	2				2				4
Total	378	155	147	70	142	57	234	56	1,239
